# S10-5 Legislations and regulations of French public policies promoting health-enhancing physical activity at the local level: do governments adopt a whole system approach?

**DOI:** 10.1093/eurpub/ckad133.052

**Published:** 2023-09-11

**Authors:** Antoine Noel Racine, Aurelie Van Hoye, Anne Vuillemin

**Affiliations:** Université Côte d’Azur, LAMHESS; University of Lorraine, APEMAC; University of Limerick, Physical Activity for Health Research Cluster; Université Côte d’Azur, LAMHESS

## Abstract

**Purpose:**

Physical inactivity are complex problems to address. System thinking approach (STA) can be a solution to take into account the diversity of upstream factors by identifying leverage points and potential co-benefits of public policies. STA has not been explored at the local level in France. Based on existing local legislations and regulations, this study aims at providing a system approach to health-enhancing physical activity (HEPA) for policymakers.

**Methods:**

This study was conducted in five stages by the research team: (1) a systematic search of document has been used to identify French public policies’ legislations and regulations promoting HEPA at local level ; (2) main characteristics for each legislation or regulation were reviewed and extracted to a data extraction form: administration holder, sector holder, political purpose, political commitment, policy timeframe and potential policy partners ; (3) a whole system map of legislations and regulations options to promote HEPA at local level was developed using STA ; (4) the HEPA policies of 10 French mid-size municipalities were collected using a local policy audit tool (i.e. CAPLA-Santé) ; (5) the system map developed was compared with the policy audit.

**Results:**

A total of 26 legislations and regulations options to promote HEPA at local level were identified from sport, education, transport, urban-planning, sustainable development, housing, social and health sectors. The comparison of the whole system map with the policy audit of mid-size municipalities showed that local governments used few legislations and regulations options among their possibilities to promote HEPA (i.e. range of 0-4).

**Conclusions:**

HEPA promotion could be integrated in many public policies at local level. However, local governments included in our study did not adopt a whole system approach to promote HEPA, rather a silo sectorial approach. The use of STA may help policymakers to highlight and develop their legislations and regulations options to promote HEPA and maximize co-benefits.

